# Assessment of Antioxidant, Immunomodulatory Activity of Oxidised Epigallocatechin-3-Gallate (Green Tea Polyphenol) and Its Action on the Main Protease of SARS-CoV-2—An In Vitro and In Silico Approach

**DOI:** 10.3390/antiox11020294

**Published:** 2022-01-31

**Authors:** Ramakrishna Ungarala, Manne Munikumar, Sukesh Narayan Sinha, Dileshwar Kumar, R. Shyam Sunder, Suresh Challa

**Affiliations:** 1 Food Safety Division, ICMR- National Institute of Nutrition, Tarnaka, Hyderabad, Telangana 500007, India; uv.ramakrishna@gmail.com (R.U.); dillu1991@hotmail.com (D.K.); 2 Clinical Division, ICMR- National Institute of Nutrition, Tarnaka, Hyderabad, Telangana 500007, India; mannemunikumar.bioinfo@gmail.com; 3 University College of Technology, Osmania University, Tarnaka, Hyderabad, Telangana 500007, India; rachamallass@yahoo.co.in; 4 Cell Biology Division, ICMR- National Institute of Nutrition, Tarnaka, Hyderabad, Telangana 500007, India; sureshnin2000@gmail.com

**Keywords:** EGCG, oxidised EGCG, antioxidant, inflammatory, molecular docking, SARS-CoV-2

## Abstract

Owing to the instability of Epigallocatechin Gallate (EGCG), it may undergo auto-oxidation and form oxidised products or dimers. In the present study, we aimed to evaluate the therapeutic effects, including antioxidation and immunomodulatory action, of the Oxidised Epigallocatechin Gallate (O-EGCG) as compared to native EGCG and the action of these compounds on main protease (M^pro^) docking against SARS-CoV-2. HCT-116 (Human Colon Cancer) cell lines were used to estimate the total antioxidant capacity and lipid peroxidation levels and pro-inflammatory markers (human IL-6, IL-1β, TNF-α). Further, molecular docking analysis was performed by AutoDock and visualised in Discovery studio. Improved antioxidant capacity of O-EGCG was observed, and there was a significant decrease in the inflammatory markers (IL-1β, IL-6, and TNF-α) when O-EGCG was applied as compared to EGCG. The O-EGCG was shown to be strongly associated with the highest docking score and active site residues of IL-1, IL-6, and TNF- α, as well as the M^pro^ of SARS-CoV-2, according to in silico approach. The in vitro and in silico analyses indicate an improved therapeutic action of the oxidised form of EGCG. The effective inhibitory action of O-EGCG against SARS-CoV-2 suggests further exploration of the compound against COVID-19 and its efficacy. However, in vivo studies and understanding of the mechanism of action of O-EGCG may yield a better opinion on the use of O-EGCG and future human clinical trials.

## 1. Introduction

Polyphenols are bioactive chemicals that contribute to the colour, flavour, and pharmacological actions of fruits and vegetables [1]. In addition to their ability to scavenge free radicals, polyphenols also have anti-inflammatory properties because they prevent the activation of important cell signalling pathways that lead to systemic inflammation [2]. The polyphenols present in green tea, made from the leaves of the *Camellia sinensis* plant, have a wide range of therapeutic properties. Green tea polyphenols (GTPs), particularly (−)-epigallocatechin-3-gallate (EGCG) and (−)-epicatechin gallate (ECG), can boost immune function and reduce the risk of inflammation and immunological disorders [3].

Among the GTPs, the catechin EGCG is found to be the most abundant compound and exhibits the highest biological activities. The antioxidant activity of EGCG has been well documented in the literature due to its ability to scavenge free radical ions and increase antioxidant enzyme activity [4]. In an animal model study, EGCG increased antioxidant enzyme levels such as superoxide dismutase and catalase and decreased protein carbonyl levels [5]. Additionally, EGCG administration has been shown to protect patients with contrast-induced nephropathy by reducing apoptosis and oxidative stress, as well as inflammation [6]. Further, the immunomodulatory role of EGCG has also been well documented as an inhibitor of cyclooxygenase-2 (COX-2) and inducible nitric oxide synthase (iNOS), and EGCG may suppress macrophage production of tumor necrosis factor (TNF-α), interleukin-1-beta (IL-1-β), and interleukine-6 (IL-6) by repressing the expression of these cytokines [1]. TNF-α and IL-6 play a significant role in the human pathophysiology of inflammation. Inflammation is a condition that causes swelling, redness, heat, soreness, and pain in the body as a result of damaged tissue or organ [7]. Furthermore, it has been linked to diseases such as allergies, atherosclerosis, arthritis, and auto-immune diseases [8].

**Figure 1:**
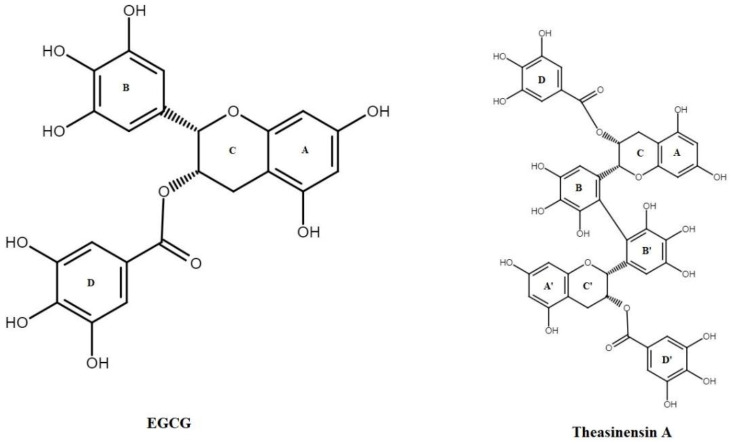
Structure of EGCG and theasinensin A.

However, EGCG may undergo various transformations, including oxidation, affecting its stability and forming dimers [9]. The chemical structure of EGCG contributes to its highly reactive properties. Since the B-ring trihydroxy group is highly active and is the primary site for antioxidant reactions, EGCG is vulnerable to oxidation in air at neutral or, especially, alkaline pH. In the process of auto-oxidation, EGCG yields a large number of EGCG auto-oxidation products (EAOPs), including reactive oxygen species [10]. The experimental study by Tanaka et al. (2003) [11] demonstrated that, upon auto-oxidation, EGCG may undergo dimerization and form theasinensins A. Further, the review by Weerawatanakorn et al. (2015) [12] discussed the possible mechanism of dimerization of EGCG molecule and formation of theasinensins A (Figure 1).

Research has suggested that it is possible for EGCG to undergo auto-oxidation, resulting in a variety of different EAOPs. To minimize cancer cell invasion and resistance to chemotherapeutic therapies, substances with the ability to deplete extracellular cystine may be useful. EGCG has the ability to covalently couple cystine residues. EGCG auto-oxidation is required for the covalent attachment of cystine residues to EGCG. This leads to the creation of ortho-quinones at the B or D ring of EGCG through auto-oxidation. Sulfhydryl groups can be conjugated with EGCG using ortho-quinones [10] as a result of its oxidative products. The antioxidant activity of theasinensins A was reported by Hashimoto et al. (2003) [13]. In the evaluation of a five-day lipid peroxidation as a trial, it was discovered that the antioxidant activity was greater than that of alpha-tocopherol (nature’s most potent fat-soluble antioxidant). Anti-inflammatory effects may be achieved by modulating the relevant expression networks for interleukins, chemokines, and interferon in the dimer form of EGCG, known as theasinensin A [14]. Hisanga et al. (2014) [15] found that theasinensin A significantly reduced the levels of pro-inflammatory mediators such as inducible nitric oxide synthase (iNOS), nitric oxide (NO), interleukin-12 (IL-12) (p70), tumour necrosis factor alpha (TNF-α), and MCP-1. These studies show that the oxidized products of EGCG have a greater therapeutic effect.

EGCG, a polyphenol found in green tea, has been shown to inhibit the 3CL protease of SARS-CoV-2, although its effect on coronavirus replication is unknown. Researchers employed HcoV-OC43 (beta) and HcoV-229E (alpha) coronavirus to study the effects of EGCG on the coronaviruses through this study. The 3CL protease activity of HcoV-OC43 and HcoV-229E is reduced by EGCG treatment. Furthermore, EGCG therapy reduced cytotoxicity caused by HcoV-OC43. The results of our study show that EGCG therapy reduces the amounts of coronavirus RNA and protein in infected cell cultures. Their research shows that EGCG reduces the replication of coronavirus [16]. Researchers found that hesperidin, nabiximols, pectolinarin, EGCG, Rhoifolin, and epigallocatechin gallate (EGCG) showed higher binding free energies with the M^pro^ and S protein of SARS-CoV-2. Although the findings of molecular docking of kaempferol, herbacetin, eugenol, and 6-shogaol are not quite as good as those chemical compounds, oral availability and Ro5 criteria are met by these substances, which have good molecular docking results. Antiviral phytochemicals, such as those found in these compounds, may be able to halt the spread of the virus [17]. Hence, EGCG and its oxidised compound could be a novel compound in acting against the replication of the virus and aid in the treatment of COVID-19. Interestingly, the most prominent immunity change occurring in COVID-19 patients indicates the elevation of these pro-inflammatory cytokines, including TNF-α, IL-1β, and IL-6, causing the prevalent cytokine shift, also called the cytokine storm, in patients with severe or terminal disease condition [18]. Therefore, this calls for an evaluation of the antioxidant and immunomodulatory effects of the oxidised product of EGCG and comparison with the native EGCG. Hence, in the present study, we attempted to oxidise the EGCG in a laboratory setup and analyse the antioxidant activity and the levels of pro-inflammatory cytokines on human colon cancer cell lines and their activity on M-Protease of SARS-CoV-2. However, since handling SARS-COV-2 cells requires high-level facilities and safety settings, we have conducted in vitro experiments on uninfected SARS-COV-2 cells. Therefore, to assess the oxidative stress and inflammation, we performed in vitro study on cancer cell lines (HCT-116), which also induces strong oxidative stress, and in silico analysis was performed on SARS-CoV-2 main protease.

## 2. Materials and Methods

### 2.1. Cell Culture and Cell Lines

HCT-116 (human colon cancer cell lines) were obtained from The National Centre for Cell Science (NCCS), Pune, India. Cells were maintained in Dulbecco’s Modified Eagle Medium (DMEM), +10% Fetal bovine serum (FBS), and antibiotics (pen-strep) until confluence at 37 °C, 5% CO_2_, and 95% relative humidity in a CO_2_ incubator.

### 2.2. Chemicals and Kits

Dulbecco’s Modified Eagle Medium (DMEM) (D6171) was purchased from M/s Sigma–Aldrich, St. Louis, MO, USA. FBS (16000-044) and Pen-strep (15140122) were obtained from Gibco Chemicals, Secunderabad, India. MTT reagent (33,611) was procured from Sisco Research Laboratorie, Mumbai, India. An antioxidant assay kit (709,001) was purchased from Cayman Chemicals, Ann Arbor, MI, USA. Human IL-6 (KB1068), IL-1β (KB1063), and TNF-α (KLU0003) ELISA kits were procured from Krishngen Biosystems, Mumbai, India.

### 2.3. Oxidation of EGCG

One gram of EGCG was dissolved in 25 mL of water and 5 mL of H_2_O_2_ (30%) was added to the solution and left at room temperature for four days, until complete oxidation was achieved and confirmed by monitoring it on HPLC [19].

### 2.4. Cell Viability Assay

The synthesized and standard compound cell viability was performed against the cancer cell line; HCT-116 was determined by using MTT assay. In 96 well microplate culture, 2.5 × 10^5^ cells were seeded and incubated at 37 °C for 24 h. After incubation, the cells were treated with different concentrations of compound (10–100 µM) in each well for 24 h incubation. At the end of exposure, the 20 μL MTT reagent (Sisco Research Laboratorie, Mumbai, India, 33,611) was added to each well, and plates were incubated for 2 h. Carefully, the supernatant was removed, formazan crystals were dissolved in 70 μL DMSO, and absorbance was recorded at 570 nm with a Spectramax microplate reader.

### 2.5. Antioxidant Activity

#### 2.5.1. Total Antioxidant Assay

HCT-116 cells were cultured in T25 flasks and treated with both the compounds upon reaching 95% confluence for 24 h, and then the cells were collected by scrapping with a rubber scrapper. At a temperature of 4 °C, the cells were centrifuged for 10 min at 1000–2000× *g*. A 1–2 mL, cold buffer was used to homogenise the cell pellet (i.e., 5 mM potassium phosphate, pH 7.4, containing 0.9 percent sodium chloride and 0.1 percent glucose). For 15 min at 4 °C, we centrifuged at 10,000× *g*. For testing, the supernatant was removed. The procedure given in the assay protocol was followed for estimation of Trolox to indicate the antioxidant activity of both EGCG and O-EGCG. In each of the Trolox Standard wells, 10 μL of Trolox standard (tubes A–G), 10 μL of Metmyoglobin, and 150 μL of Chromogen were added to the designated wells on the plate. Metmyoglobin and chromogen were added to two sample wells, each containing 10 microliters of sample and 10 microliters of metmyoglobin. To initiate the reactions, 40 μL of hydrogen peroxide working solution was added to each of the wells. It was incubated on a shaker at room temperature by sealing the plate for 5 min, and absorbance was measured at 750 nm or 405 nm. In order to determine the antioxidant concentration, we used the equation shown below.

(1) $$\mathrm{Antioxidant}\;(\mathrm{mM})=\left[\frac{\left(sample\;average\;absorbance\right)-\left(Y-intercept\right)}{Slope}\right]\times \;Dilution$$

#### 2.5.2. TBARS Assay

Thiobarbituric acid reactive species (TBARS) have been measured as an estimate of global oxidative stress levels (lipid peroxidation) in cell lysate of untreated and treated cells with EGCG and O-EGCG. Levels of malondialdehyde (MDA)—a by-product of lipid peroxidation of thiobarbituric acid (TBA)—were estimated by colorimetric measurement using 1,1,3,3-Tetramethoxypropane (TMP) standard. Briefly, 100 µL of cell lysate was mixed thoroughly with 10% trichloroacetic acid (TCA) and freshly prepared 0.67% TBA and heated at 90 °C for 30 min in a water bath, cooled to RT, and centrifuged. Upon addition to n-butanol and vigorous shaking, MDA formed (pink-coloured product) in the organic layer was measured at 532 nm (cell lysate), and concentrations were computed from the standard curve, with values represented as mean ± SE from three independent experiments performed in duplicates.

### 2.6. Cell Supernatant Collection

Cells were cultured in T-25 flasks and, upon reaching 95% confluence, the flasks were divided into Untreated, EGCG Treated, and O-EGCG Treated with IC-10, IC-25, and IC-50 concentration and left for 24 h. Cell culture supernatant containing secrete components, i.e., media was collected into sterile tubes and centrifuged at 2000–3000 RPM for 20 min and the supernatants were collected.

### 2.7. Inflammatory Markers

#### 2.7.1. Human IL-6

Human IL-6 GENLISA ELISA KIT was procured from Krishngen Biosystems, Mumbai, India, and the procedure mentioned in the kit protocol was followed for the assay. Approximately 100 μL of standards and samples were added to the plate. The assay diluent (1×) acts as a zero standard (0 pg/mL). At room temperature (18–25 °C), the plate was sealed and incubated for 2 h. Each well was aspirated and washed four times with wash buffer (1×), followed by the addition of 100 μL diluted detection antibody solution. At room temperature (18–25 °C), the plate was sealed and incubated for 1 h. After four washes with wash buffer (1×), each well was filled with 100 μL diluted streptavidin–HRP solution. For 30 min at room temperature (18–25 °C), the plate was sealed and incubated. Additionally, the plate was washed four times with wash buffer (1×) and 100 μL TMB substrate solution was added and incubated at room temperature for 15–30 min in the dark. The colour of the positive wells changed to a bluish hue. Each well received 100 μL of stop solution. Within 30 min of stopping the reaction, positive wells turned yellow and the absorbance at 450 nm was determined.

#### 2.7.2. Human IL-1β

Human IL-1β GENLISA ELISA KIT was procured from Krishngen Biosystems, Mumbai, India, and the procedure mentioned in the kit protocol was followed for the assay. A 100 μL dose of standards and samples was added to the plate. The assay diluent (1×) was used as the reference standard (0 pg/mL). The plate was sealed and incubated at 37 °C for two hours. The plate was aspirated and washed four times with wash buffer (1×), and 100 μL diluted detection antibody solution was added to each well. The plate was then sealed and incubated at 37 °C for 1 h. The plate was washed four times with wash buffer (1×), and each well was filled with 100 μL diluted streptavidin–HRP solution. The plate was then sealed and incubated at 37 °C for 30 min. Additionally, the plate was washed four times with wash buffer (1×), and 100 μL TMB substrate solution was added and incubated in the dark at 37 °C for 15–30 min. Positive wells developed a bluish hue. Each well received 100 μL of stop solution. Within 30 min of stopping the reaction, the positive wells turned yellow and the absorbance at 450 nm was measured.

#### 2.7.3. TNF-α

Human TNF-α GENLISA ELISA KIT was procured from Krishngen Biosystems, Mumbai, India, and the procedure mentioned in the kit protocol was followed for the assay. A total of approximately 100 μL of standards and samples was added to the plate. The assay diluent (1×) was used as the reference standard (0 pg/mL). The plate was sealed and incubated at 37 °C for two hours. The plate was aspirated and washed four times with wash buffer (1×), and 100 μL diluted detection antibody solution was added to each well. The plate was then sealed and incubated at 37 °C for 1 h. The plate was washed four times with wash buffer (1×), and 100 μL diluted streptavidin–HRP solution was added to each well. The plate was then sealed and incubated at 37 °C for 30 min. Additionally, the plate was washed four times with wash buffer (1×), and 100 μL TMB substrate solution was added and incubated in the dark at 37 °C for 15–30 min. Positive wells developed a bluish hue. Each well received 100 μL of stop solution. Within 30 min of stopping the reaction, the positive wells turned yellow and the absorbance at 450 nm was measured.

### 2.8. In Silico Molecular Docking

We retrieved co-crystal structures of human IL-1 in association with its receptor 1L-1R (PDB ID:1ITB) [20,21], human interleukin-6 with L(+)-tartaric acid (PDB ID:1ALU) [22], human tumour necrosis factor-alpha (2az5) [23], and main protease of SARS-CoV-2 with N-[(5-Methylisoxazol-3-Yl)Carbonyl]Alanyl-L-Valyl-N~1~-((1r,2z)-4-(Benzyloxy)-4-Oxo-1-{[(3r)-2-Oxopyrrolidin-3-Yl] Methyl}But-2-Enyl)-L-Leucinamide (N3 peptide) (PDB ID:6LU7) [24] from Protein Data Bank (PDB: https://www.rcsb.org/, accessed on 20 November 2021). By exploring the Auto Dock Vina 1.5.6 software (The Scripps Research Institute, La Jolla, San Diego, CA, USA), the retrieved co-crystal structure was optimised for molecular docking analysis. To initiate the molecular docking process, all water molecules and heteroatoms were removed, and polar hydrogen atoms and charges were added to the structures. The co-crystal native compounds were separated by releasing atomic coordinates of the PDB file [25]. After removing all water molecules, in order to generate the necessary files for AutoDock Vina, hydrogen polarities were assigned, Gasteiger charges were calculated for protein structures, and protein structures were converted from the PDB file format to the PDBQT format using the auto dock tool (ADT) software.

The 3D structures of EGCG (PubChem ID: 65064) and Theasinensin A (PubChem ID: 442543) were obtained from the PubChem Compound Database (National Center for Biotechnology Information; https://pubchem.ncbi.nlm.nih.gov/, accessed on 20 November 2021) in the file format of structure data file (SDF). The SDF format’s chemical structures were converted to PDB files using Discovery Studio Biovia 2020, and ADT was then used to analyse ligand structures in terms of non-polar hydrogen combinations, Gasteiger change additions, and rotatable bonds. The ligand was then translated to PDB format for molecular docking experiments, with the PDBQT format used with ADT (Version 1.5.6).

The grid spacing was set to 1.0, with a grid box size of 10 × 10 × 10 (*x*, *y*, and *z*) coordinates, and the grid centre was designed at *x*, *y*, and *z* dimensions (Supplementary Table S1) [26,27,28]. Other docking settings were left at their default parameters. For the structures, a PDBQT file was generated, which was then submitted to the molecular docking approach [29]. When the compounds interacted with macromolecules under rigid circumstances, they were in a flexible condition. To execute AutoDock Vina, Notepad was launched in order to navigate to the settings file [30,31]. Each structure contained Kollman charges and polar hydrogen atoms [32,33].

The AutoDock Vina scoring technique was used to determine negative Gibbs free energy (∆G) scores (kcal/mol) to estimate compound binding affinities. The distances between docked compounds and their interaction radii of 6 were calculated using Discovery Studio Biovia v2020 to determine the sizes of binding sites and the hydrogen bond and hydrophobic interactions they form [26,27,28,29]. For each compound’s interactions with the protein, binding poses were evaluated, and their most energetically favourable conformations were chosen [34].

### 2.9. Statistical Analysis

All results are expressed as the mean standard deviation (SD) of three independent experiments. SPSS software was used to conduct statistical analyses, which included a one-way ANOVA comparison of treated and untreated cells. A null hypothesis probability of <5% (*p* < 0.05) was considered statistically significant.

## 3. Results

### 3.1. Oxidation of EGCG

Standard EGCG was oxidized using a mild oxidizing agent, H_2_O_2_. About 200 mg of standard EGCG was dissolved in 5 mL of water. To this, a standard solution of 30% H_2_O_2_ (1 mL) was added and left at room temperature. This reaction mixture was monitored using an HPLC system to observe the reaction and check the total oxidation of EGCG. The total conversion was observed after four days of the initiation of reaction, i.e., after approximately 96 h, and we could also observe a colour change of the sample form pink to dark orange. The oxidised compound was used for further study.

### 3.2. Cell Viability Assay

**Figure 2:**
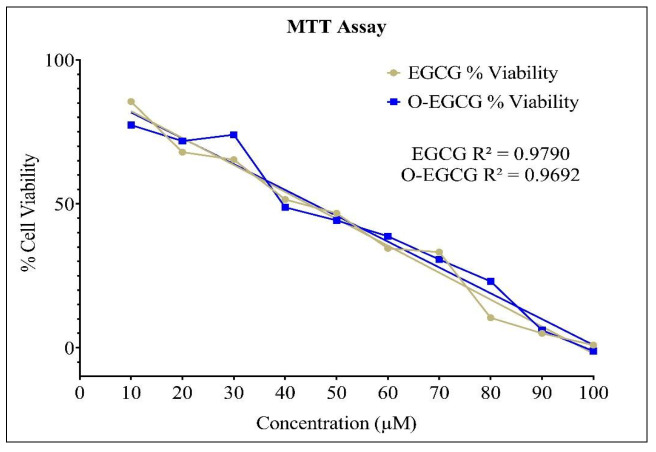
Cell viability of EGCG and O-EGCG on HCT-116 cells.

A cell viability assay as a preliminary study showed that more than 90% of HCT-116 cells were viable at 8.4 and 8.5 µM of EGCG and O-EGCG, respectively (Table 1). EGCG and O-EGCG showed cell viability at IC50 values of 44.44 µM and 46.49 µM, respectively (Figure 2).

### 3.3. Antioxidant Assay

**Figure 3:**
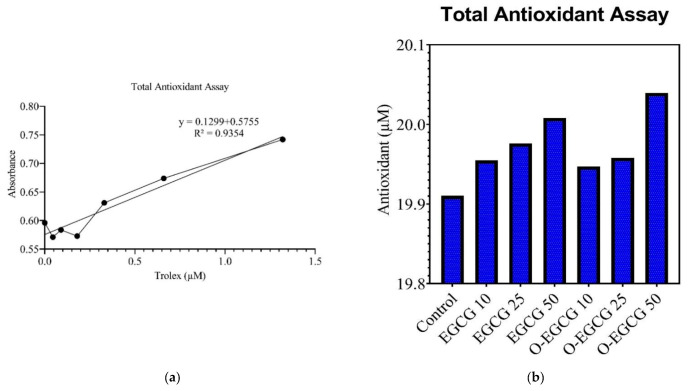
(**a**) Calibration curve of Trolox standard. (**b**) Levels of antioxidant in EGCG and O-EGCG.

The antioxidant levels in EGCG- and O-EGCG-treated HCT-116 cells were compared with the levels of control (untreated cells) observed from the assay reaction. Metmyoglobin oxidises ABTS^®^ (2,2′-Azino-di-[3-ethylbenzthiazoline sulphonate]) to ABTS^®^•+, so the assay relies on antioxidants in the sample to prevent this oxidation. The Trolox standard curve showed a linear regression of 0.935 (Figure 3a). There was a significant increase in the total antioxidant levels in both the compounds EGCG and O-EGCG of the IC-10, IC-25, and IC-50 concentrations, as compared with the control group (Table 2, Figure 3b). The oxidised compound has shown a small increase in the total antioxidant levels, as compared with EGCG.

### 3.4. Thiobarbituric Acid Reactive Species Assay (TBARS)

**Figure 4:**
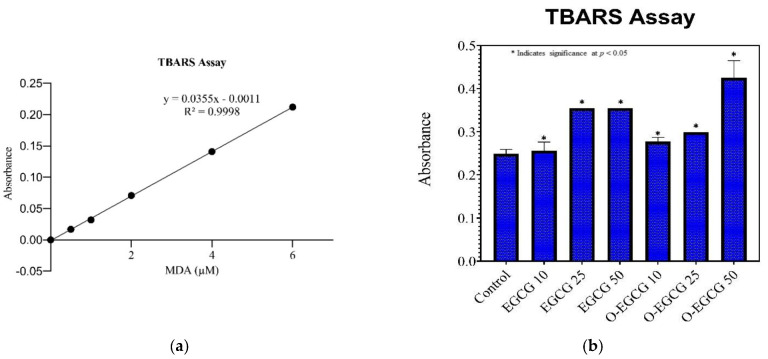
(**a**) Calibration curve of 1,1,3,3-Tetramethoxypropane. (**b**) Levels of TBARS formed by EGCG and O-EGCG. (* indicates significance at *p* < 0.05).

In order to assess the antioxidant activity of EGCG and it oxidised compound, TBARS assay was performed by estimating the levels of MDA formed by lipid peroxidation during the assay reaction process. The standard 1,1,3,3-Tetramethoxypropane (TMP) calibration curve has shown a linear regression of 0.9998 (Table 3, Figure 4a). We chose to use a range of concentrations (i.e., IC-10, IC-25, and IC-50) for EGCG and O-EGCG to observe the change in levels of MDA formed by the compounds. As shown in the Figure 4b, EGCG and O-EGCG showed a significant increase in the IC10–IC50 levels of MDA formed, as compared with the control group.

The results obtained by the TBARS assay are similar to the results of the total antioxidant activity, and show a significant increase in all concentrations of both of the compounds, as compared with the control group. A small increase in the antioxidant levels were observed in the IC50 of the oxidation product as compared to EGCG, indicating better antioxidant activity.

### 3.5. Effect of EGCG and O-EGCG on Inflammatory Markers

Given the antioxidant enzyme levels and their reduction, which is a key indicator for inflammatory response, we measured the levels of cytokines as IL-6, IL-1β, and TNF-α within HCT 116 cells using ELISA techniques. The cells were treated with IC-10, IC-25, and IC-50 concentrations of EGCG and its oxidative product (O-EGCG), then compared with an untreated control group.

### 3.6. Human IL-1β

**Figure 5:**
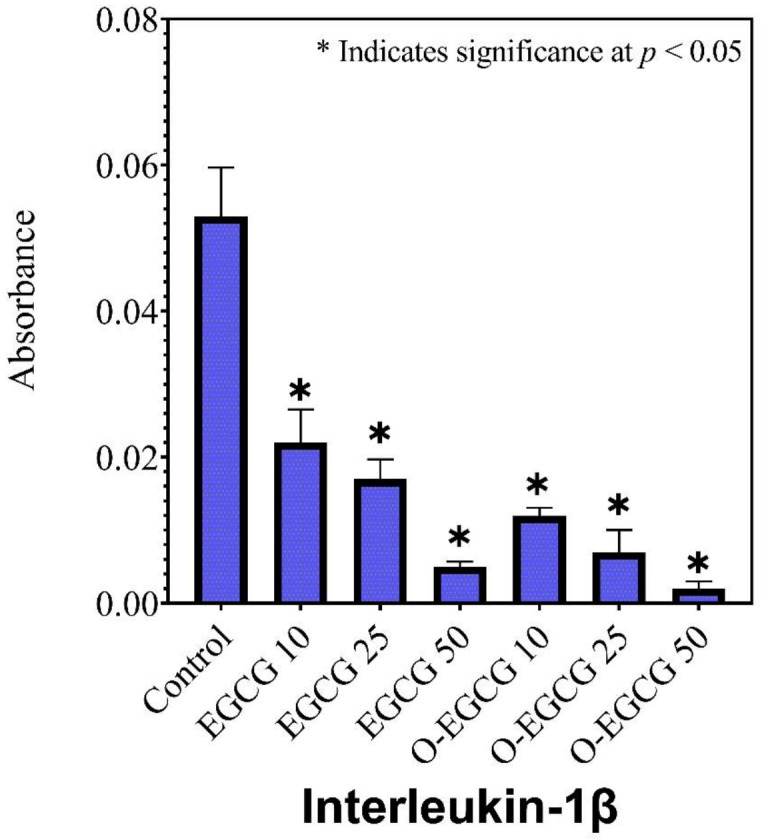
The effect of EGCG and O-EGCG on IL-β production in HCT-116 cells. (* indicates significance at *p* < 0.05).

IL-1beta is a pro-inflammatory cytokine and belongs to the IL-1 family. The cytokine interleukin-1 beta (IL-1) is an important factor in the activation of inflammatory processes. IL-1 beta and COVID-19:IL-1 beta are known to play a central role in cytokine release syndrome (CRS). Excess IL-1 beta causes a cytokine storm, increasing fatality in patients. Compared to the control group, all concentrations of EGCG and its oxidised form showed a significant decrease in IL-1 beta protein levels (Table 4, Figure 5). It was found that the oxidised form of EGCG had a greater ability to reduce inflammation than the non-oxidized form of EGCG.

### 3.7. Human IL-6

**Figure 6:**
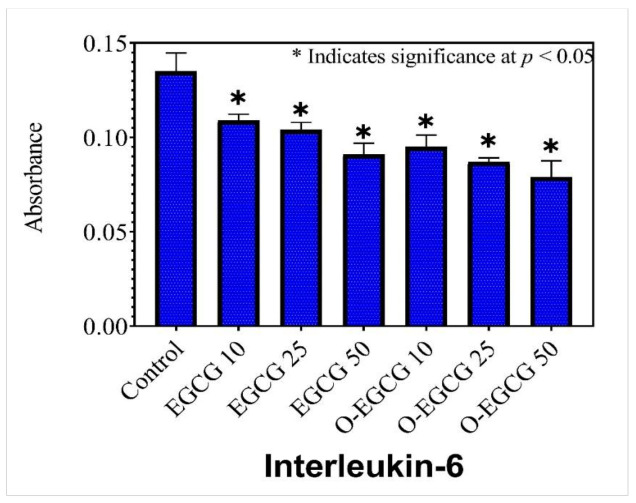
The effect of EGCG and O-EGCG on IL-6 production in HCT-116 cells. (* indicates significance at *p* < 0.05).

Interleukin-6 (IL-6) is a member of the IL-6 family. Acute-phase reactions are induced by IL-6. These help to activate innate immunity, which helps to prevent tissue damage. It also aids in the early differentiation of T-cells. A significant decrease in the levels of IL-6 proteins was observed in all the concentrations of EGCG and its oxidised form, as compared with the control group (Table 4, Figure 6). A further decrease was observed in the IC-50 concentration of the oxidised form than in that of EGCG, indicating the greater ability to reduce inflammation.

### 3.8. TNF-Alpha

**Figure 7:**
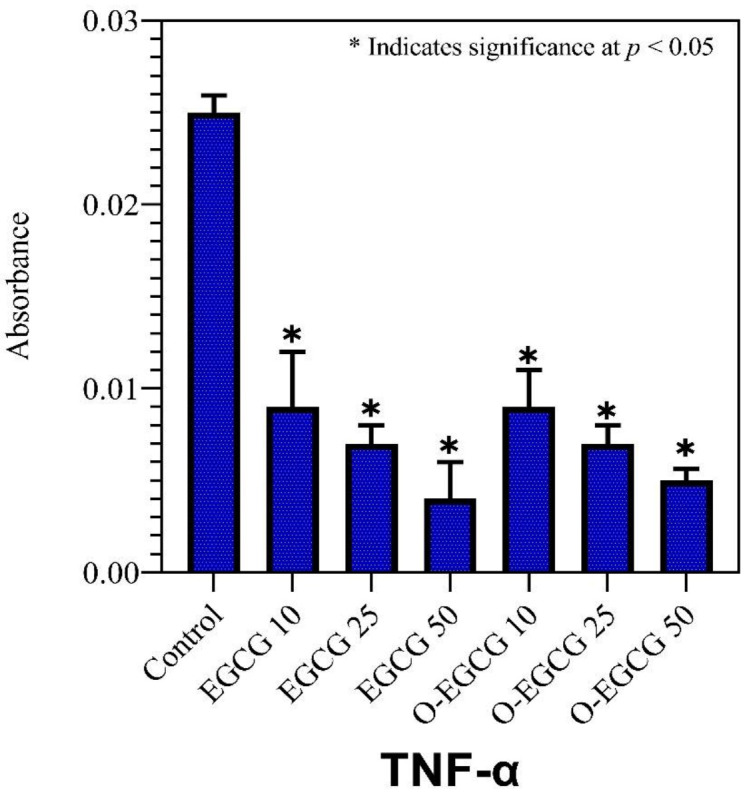
The effect of EGCG and O-EGCG on TNF-α production in HCT-116 cells. (* indicates significance at *p* < 0.05).

Inflammation is a critical component of innate immunity, and it is regulated through a number of different mechanisms. In the cytokine network, TNF-α is a key player in regulating this process and is a potent paracrine and endocrine mediator of inflammatory and immune functions. As compared to the control group, EGCG and its oxidised form significantly reduced the levels of TNF-α proteins in all concentrations of EGCG and O-EGCG (Table 4, Figure 7).

### 3.9. In Silico Analysis

#### 3.9.1. Anti-Inflammatory and Pro-Inflammatory Analysis

According to Viger et al., 1997 and 2000, the IL-1R binding site is dissected into two sub-sites, A (IL-1α) and B (IL-1β) [20,21]. Both the sites interacted with distinct domains of the IL-1R receptor via site-A residues of Arg-11, Gln-14, Gln-15, Lys-27, Gln-32, Gly-33, Gln-34, and Glu-128 and site-B of Ala-1, Agr-4, Glu-51, Lys-93, Lys-94, Glu-105, and Asn-108 [20]. These interaction sites play a significant role in receptor binding and signal induction. As a result, they might serve as targeted locations for compound binding, and the residues were identified for the active site for molecular docking analysis [35].

**Figure 8:**
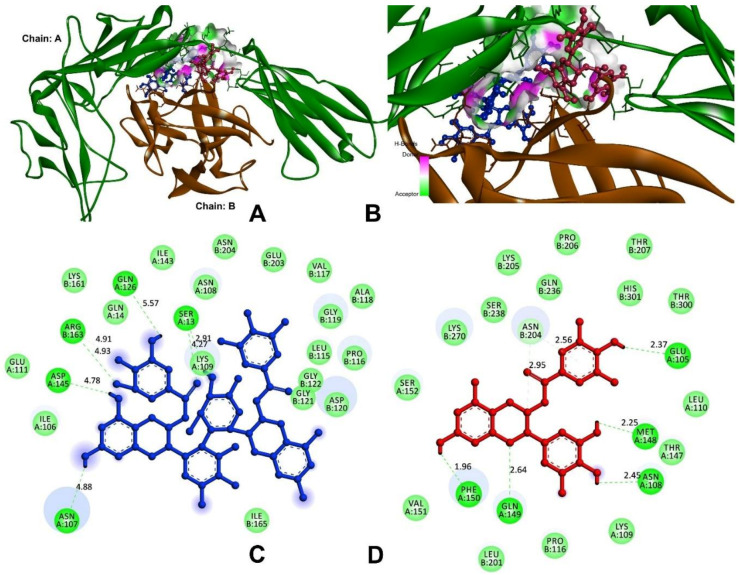
Molecular docking and interactions of IL-1 with theasinensin A and epigallocatechin gallate. (**A**) Chain-A (**green**) and chain-B (**brown**) of IL-1 bound with compounds of theasinensin A (**blue**) and epigallocatechin (**red**). (**B**) higher magnification of theasinensin A (**blue**) and epigallocatechin gallate (**red**). (**C**) Molecular interactions of IL-1 and theasinensin A (**blue**). (**D**) Molecular interactions of IL-1 and epigallocatechin (**red**).

In order to block the receptor binding, signal induction, and interaction of IL-1β with IL-1R, the compounds of theasinensin A and EGCG were interacted with the cleft of site-A and B sites (Figure 8). The theasinensin A had the lowest docking score of −8.9 kcal/mol and formed seven hydrogen bonds with IL-1β and IL-1R complex (Table 5). The Ser13 (2) of site-A residues produced two h-bonds; Asn107, Gln126, and Asp145 formed the remaining five h-bonds, and Arg163 (2) of site-B residues formed two h-bonds within the range of 4 Å of the theasinensin A. While epigallocatechin gallate has a docking score of −8.3 kcal/mol and has established seven h-bonds with the site-A and site-B complexes, further EGCG formed the first binding site-A residues of Glu105, Asn108, Met148, Gln149, and Phe150; in addition, the binding site (B) formed two residues of Asn204.

**Figure 9:**
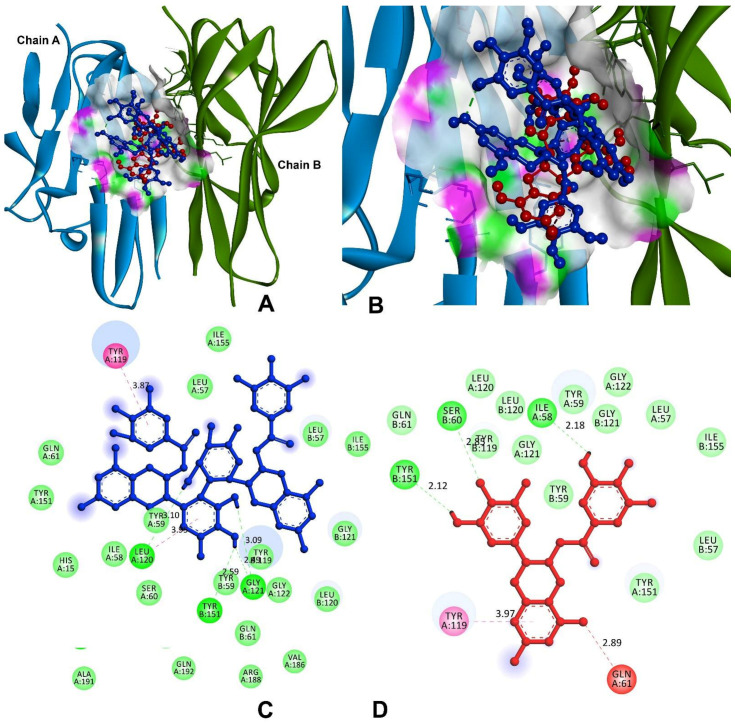
Molecular docking and interactions of TNF-α with theasinensin A and epigallocatechin gallate. (**A**) Chain-A (**Cyan**) and chain-B (**green**) of TNF-α bound with compounds of theasinensin A (**blue**) and epigallocatechin (**red**). (**B**) Higher magnification of theasinensin A (**blue**) and epigallocatechin gallate (**red**) with TNF-α. (**C**) Molecular interactions of IL-1 and theasinensin A (**blue**). (**D**) Molecular interactions of TNF-α and epigallocatechin (**red**).

Molecular docking studies were carried out on theasinensin A and EGCG, both molecules that have shown promising anti-inflammatory potential in vitro, along with co-crystal ligand (native) of 6,7-dimethyl-3-[(methyl{2-[methyl({1-[3-(trifluoromethyl)phenyl]-1h-indol-3-yl}methyl) amino]ethyl}amino)methyl]-4h-chromen-4-one as a standard towards protein target 2AZ5 (TNF-α) [36]. The native small molecule compound was bound with TNF-α homomer chains A and B (Figure 9). The chain-A residues bound with Leu57, Tyr59, Ser60, Gln61, Tyr119, Leu120, Gly121, Gly122, and Tyr151, while the remaining seven are a subset of these residues present in the chain-B of Leu55, Tyr59, Ser60, Tyr119, Leu120, Gly121, and Tyr151. Among the tested compounds, theasinensin A showed the highest (−7.2 kcal/mol) docking score, while this value was lowest (−6.4 kcal/mol) (Table 5) for epigallocatechin gallate against TNF-α. The theasinensin A formed six h-bonds with chain-A (5) and chain-B residues of Tyr119, Leu120, Leu120, Gly121, Gly121, and Tyr151, respectively, while epigallocatechin gallate formed five with chain-A of Ile58, Gln61, and Tyr119 and chain-B of Ser60 and Tyr151.

**Figure 10:**
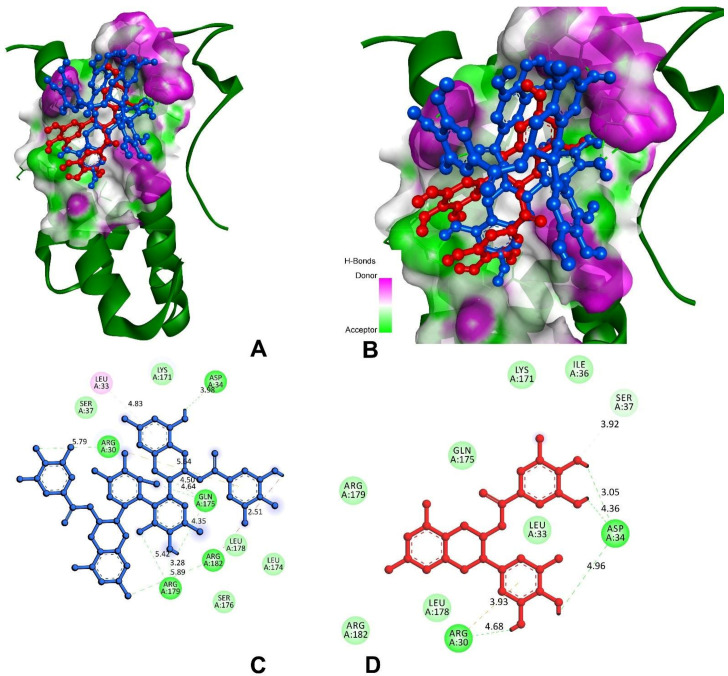
Molecular docking and interactions of IL-6 with theasinensin A and epigallocatechin gallate. (**A**) IL-6 (**green**) bound with compounds of theasinensin A (**blue**) and epigallocatechin (**red**). (**B**) Higher magnification of theasinensin A (**blue**) and epigallocatechin gallate (**red**) with IL-6. (**C**) Molecular interactions of IL-6 and theasinensin A (**blue**). (**D**) Molecular interactions of IL-6 and epigallocatechin (**red**).

In the case of IL-6, the compounds of theasinensin A (−7.2 kcal/mol) and EGCG (−6.4 kcal/mol) (Table 5) showed the highest and lowest molecular docking score values, respectively. Interactive h-bonds between the protein and compounds are shown in Figure 10. The IL-6 target protein formed four h-bonds with Arg179, three h-bonds with Gln175, and two each of h-bonds with Arg30 and Arg182 were found in the theasinensin A. The EGCG formed six h-bonds, two with Arg30 and three with Asp34, and one with Ser37.

#### 3.9.2. Inhibitor against Main Protease of SARS-CoV-2

**Figure 11:**
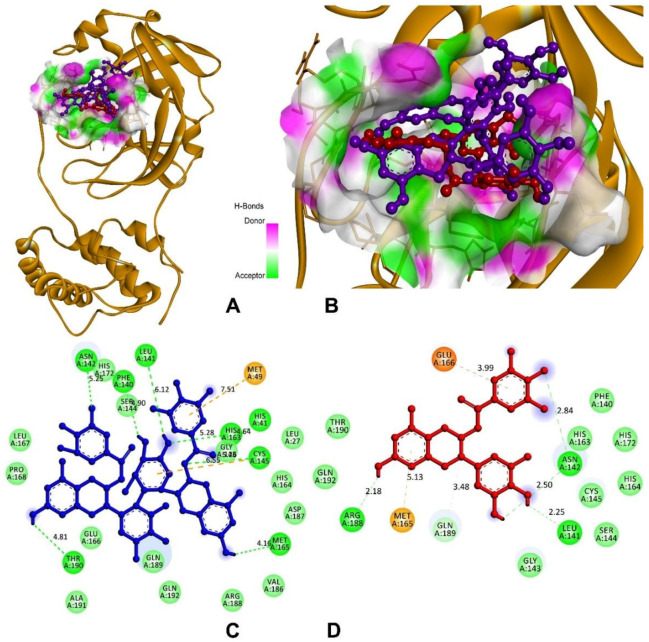
Molecular docking and interactions of M^pro^ with theasinensin A and epigallocatechin gallate. (**A**) M^pro^ (**yellow**) bound with compounds of theasinensin A (**blue**) and epigallocatechin (**red**). (**B**) Higher magnification of theasinensin A (**blue**) and epigallocatechin gallate (**red**) with M^pro^. (**C**) Molecular interactions of M^pro^ and theasinensin A (**blue**). (**D**) Molecular interactions of M^pro^ and epigallocatechin (**red**).

3CL protease, or M^pro^, facilitated the maturation of functional polypeptides involved in the assembly of replication–translation machinery [37]. M^pro^ begins by cleaving this enzyme from pp1a and pp1ab, then proceeds to cleave the polyprotein at 11 conserved locations [38]. The molecular docking analysis and visualisation of M^pro^ binding with theasinensin A and EGCG is shown in Figure 11. The compound theasinensin A exhibits the best docked score (−8.70 kcal/mol) with SARS-CoV2 M^pro^ (Table 5) and molecular interactions with the residues of His41, Met49, Phe140, Leu141, Asn142, Cys145, His163, Met165, and Thr190. The EGCG has a binding affinity of −7.77 kcal/mol with M^pro^ of SARS-CoV-2. Leu141, Asn142 (2), Met165, Glu166, Arg188, and Gln189 were the residues participating in the interaction at the binding pocket of the main protease of SARS-CoV2.

## 4. Discussion

The indication of auto-oxidation of EGCG and formation of dimers led to the present study, which investigated the therapeutic activity of EGCG and its oxidized product. Human colon cancer cell lines were used to investigate the antioxidation and immunomodulatory action of EGCG and O-EGCG. With the intriguing results obtained in in vivo studies, we attempted to conduct an in silico analysis to analyse the anti-inflammatory and pro-inflammatory effect of EGCG and O-EGCG and their inhibitory action against the main protease of SARS-CoV-2.

When examined on various cell lines, including MCF-7, SV-80, HepG2, Y-79, and Caco-2, EGCG exhibited lower cell viability [39]. EGCG treatment reduced cell viability in Caco-2, MCF-7 (10 M yielded 70% viability [40]), and HepG2 (IC50 at 48 h exposure was 74.7 g/mL), according to other researchers [41].

The most likely possible markers for EGCG antioxidant responses in biological systems are oxidation products of the B-ring of EGCG. To further understand how antioxidant reactions affect catechins’ ability to protect against disease, further investigation of these compounds may be recommended [42]. EGCG can be chemically altered to change its relative therapeutic activity, allowing for synergistic supplementation to improve health benefits [43]. The total phenolic and flavonoid content of plant extracts was found to have a positive linear connection with antioxidant activity. In addition to their anti-inflammatory properties, plant extracts high in phenolic and flavonoid content also showed good cell survival [44]. The oxidation product has shown an increase in antioxidant activity as compared to the reduced form, which makes it a promising compound to show elevated anti-inflammatory responses.

One of the polyphenols in green tea, EGCG, has been shown to have the highest antioxidant capacity [45]. The galloyl groups on the B and D rings are thought to be responsible for EGCG’s radical scavenging abilities. Since EGCG reacts with O_2_−, leading to oxidation of the D ring−, the vicinal trihydroxy group in the B-ring is the primary site responsible for antioxidant reactions, which are further strengthened by the vicinal trihydroxy group in the D-ring [46,47]. Erythrocytes treated with 1 to 15 M of EGCG showed reduced levels of lipid peroxidation and ATPase damage [48], whereas UVA-irradiated keratinocytes exposed to EGCG produced less hydrogen peroxide [49]. UV-induced hydrogen peroxide and DNA damage can both be reduced using green tea extract. Treatment of leukocytes with EGCG reduced bleomycin-induced DNA damage, but it had no effect on DNA repair [50]. Cao et al. (2017) [51] found that EGCG inhibited apoptosis in αTC1-6 cells exposed to H_2_O_2_-induced oxidative stress. They further believe that EGCG’s positive effects on αTC1-6 cells are due to its activation of Akt and inhibition of PARP, caspase-3, P38, and JNK MAPK. Furthermore, a study by Luo and colleagues in 2021 [52] demonstrated that EGCG had an anti-proliferative effect on SW480, SW620, and LS411N colorectal cancer cells by down-regulating the level of STAT3. Experimentation on the effectiveness and safety of EGCG as an anti-cancer supplement for people with colon cancer has been suggested following an examination into its effectiveness in animals and humans with colon cancer. Additionally, the anti-inflammatory properties of EGCG, particularly on human primary T cells, are well-documented. A recent study by Huang et al. (2021) [53] showed that EGCG regulates IL-2 and TNF-alpha levels, while Th1 cells producing inflammatory cytokines, TNF-alpha, and IL-2 showed that they can be inhibited during inflammation. According to the findings, EGCG has the ability to effectively decrease the release of cytokines by activated human primary T cells. Additionally, EGCG is known to exhibit anti-inflammatory effects, especially stimulating the human primary T cells.

The oxidized form of EGCG has shown a greater potential to exhibit antioxidant activity than EGCG, however, the oxidation products of EGCG have not been studied for their biological effects. Enzyme-catalysation and auto-oxidation are two of the various ways in which EGCG can be oxidized, as indicated in the examples above. Regardless of the oxidation method, EGCG is always polymerized, resulting in a large number of EGCG oxidation products [54]. Theasinensins are produced through the enzymatic oxidation of a commercially accessible combination of green tea catechins [55]. Gallocatechin gallate (GCG), an epimer of EGCG, was generated when the auto-oxidation speed of EGCG was prolonged by SOD in previous research, while theasinensin A, an EGCG dimer, had been reported as a temporary intermediate along with the EGCG auto-oxidation [56]. Theasinensin A (or D) and GCG were found in 2-h EAOPs by Wei et al., 2016 [10]. The antioxidant activity of theasinensins A–E was reported by Hashimoto et al. (2003) [13] to vary from 9 to 13%, in comparison to 3% and 17% for BHA and alpha-tocopherol, in a five-day lipid peroxidation. It appears that theasinensins A–E have a lesser lipid oxidation inhibitory activity than BHA (the synthetic antioxidant), but a stronger lipid oxidation inhibitory activity than alpha tocopherol.

Despite the paucity of research on EAOPs and their anti-inflammatory effects, theasinensins’ molecular mechanism has received little attention. Theasinensin A may have inhibited COX-2 production by down-regulating TAK1-mediated NF-κB and MAPK signalling pathways [57]. Using a genome-wide DNA microarray, researchers were able to identify the molecular mechanism of theasinensin A’s anti-inflammatory actions. It was discovered in LPS-activated RAW264 cells that the expression levels of 406 genes increased three-fold, while theasinensin A treatment decreased the signals of 259 of these genes by a factor of two (two-fold). LPS-activated cells reduced the expression of 717 genes by a factor of three, of which 471 genes were recovered by theasinensin A treatment (a two-fold increase). This suggested that theasinensin A had anti-inflammatory effects by modulating the expression networks of chemokines, interleukins, and interferons [14]. It has been shown that theasinensin A can reduce the levels of pro-inflammatory cytokines, such as inducible nitric oxide synthase (iNOS), nitric oxide (NO), interleukin-12 (IL-12), TNF- α, and MCP-1, in LPS-activated macrophages in an in vitro experiment. These findings reveal a cellular signalling pathway in which the theasinensin A directly inhibited MAPK/ERK kinase (MEK) signalling by directly binding to MEK–ERK for the inhibitory activity. Theasinensin A also reduced LPS-induced mouse paw edema and inhibited the production of IL-12 (p70), TNF-α, and MCP-1 in the in vivo research [15].

We extended the present study by exploring molecular docking analysis with the anti-inflammatory and pro-inflammatory proteins of IL-1, IL-6, and TNF-α with the compounds theasinensin A and EGCG, respectively. The complex IL-1α and IL-1β is a major mediator of signal transduction and inflammatory disease, and inhibiting this complex might be a potential pharmacological target for the development of novel therapeutic candidates. In the delivery of drugs, enzyme catalysis, and biosensors, peptides have been explored extensively as probes in protein–protein or peptide–protein interactions [58], and hence N3 peptides were studied in our docking. The compounds theasinensin A and EGCG were docked in the complex of IL-1α and IL-1β; the domain binding residues of Glu105, Asn107, Asn108, Ser13, Gln126, Asp145, Met148, Gln149, and Phe150 of IL-1α and IL-1β of Arg163 and Asn204 were potentially formed h-bonds replacing the native complex interactions that were formed between its cleft. In this instance, the native complex of TNF-α co-crystalized with the small molecule of 6,7-dimethyl-3-[(methyl{2-[methyl({1-[3-(trifluoromethyl) phenyl]-1h-indol-3-yl} methyl) amino] ethyl} amino) methyl]-4h-chromen-4-one, IL-6 with L (+)-tartaric acid, and M^pro^ of SARS-CoV-2 complexed with N3 peptide, which were also replaced with theasinensin A and epigallocatechin compounds.

The search for novel medications, particularly those derived from plants, holds enormous promise. Molecular docking by Platella et al. in 2020 [59] showed that the primary contact is of the hydrogen bonding type and, similarly, hydrogen atoms with polar charges were introduced to the structures before beginning the molecular docking procedure in our study. Stilbenoid analogues, previously identified for various biological functions, were repurposed against the SARS-CoV-2 spike protein and the human ACE2 receptor complex using molecular dynamics modelling and binding free energy analysis based on molecular docking in a study by Wahedi HM et al., 2021 [60]. Molecular docking simulations were performed on the A chain receptor-binding domain (RBD) surface as well as the exposed B chain and C chain interfaces at the base of the A chain RBD, in a study on the interference of Polydatin/Resveratrol in the ACE2, titled Spike recognition during COVID-19 infection. The molecular docking simulations were conducted around the ACE2 protease domain 1 and 2 helices. Several polar glucoses (with Gly205, Glu208, Asp206, Ala396, and Lys562) and resveratrol moiety (with N-acetylglucosamine 905, His195) interactions resulted in a high docking score (8.4 kcal/mol) [61], which was similar to our study, where the oxidised polyphenol showed higher docking scores on the treatment with the M-protease of SARS-CoV-2.

The results have reported that the compounds might block the binding site residues and elevate the levels of human IL-1, IL-6, and TNF-α inflammatory markers and also of the SARS-CoV-2, thereby effectively preventing the protein binding by the virus.

## 5. Conclusions

Overall, the present study examined the therapeutic effects, such as antioxidation and anti-inflammatory action, of EGCG and O-EGCG. Previous studies conducted on EGCG have already proven this effect. However, due to its instability, we examined the effect of O-EGCG and found that the oxidized product showed increased ability and effectiveness in both in vitro and in silico studies. The in vitro studies showed an increase in total antioxidative capacity of the O-EGCG as compared with EGCG. It was observed that EGCG and O-EGCG demonstrated a significant increase in the IC-10 – IC-50 levels of MDA formed, as compared with the control group. O-EGCG has shown a better antioxidant activity and greater ability to reduce inflammation than the non-oxidized form of EGCG. Furthermore, IL-6 proteins were significantly reduced in all the concentrations of O-EGCG, and it also significantly reduced the levels of TNF-α proteins as compared with the control group. The in silico studies of molecular docking on AutoDock Vina software of theasinensin A and EGCG were performed with the complexes of IL-1α, IL-1β chains, IL-6, and TNF-α, and the results showed a high docking score for both the compounds. The complexes potentially formed h-bonds, which were replaced in the native complex interactions, and oxidised compound has shown elevated docking scores as compared to the parent form. The docking with M protease of SARS-CoV-2 has also shown that the compounds are significantly blocking the attachment of binding sites and acting against the COVID virus. Although the present study showed the enhanced therapeutic activity of the oxidised form of EGCG, the characterization of the biological properties of EAOPs will be useful for gaining an in-depth understanding of their therapeutic action in vitro and may help gain insight into molecular mechanisms in vivo. However, due to the complexity of the polyphenols in tea, their biological actions of EAOPs have not been extensively examined due to challenges in obtaining pure molecules.

## Figures and Tables

**Table 1:** MTT assay of EGCG and O-EGCG on HCT-116 cells.

Sample (µM)	EGCG	O-EGCG
	% Viability	
10	85.55 ± 0.01	77.37 ± 0.10
20	67.97 ± 0.05	74.03 ± 0.17
30	65.36 ± 0.08	71.82 ± 0.15
40	51.52 ± 0.08	48.80 ± 0.11
50	46.74 ± 0.05	44.23 ± 0.22
60	34.51 ± 0.12	38.73 ± 0.13
70	33.25 ± 0.10	30.68 ± 0.09
80	10.42 ± 0.04	23.07 ± 0.11
90	4.99 ± 0.04	6.09 ± 0.03
100	0.91 ± 0.04	−1.15 ± 0.05

**Table 2:** Total antioxidant assay of EGCG and O-EGCG.

Trolox (µM)	Absorbance	Sample	Absorbance	Sample	Antioxidant (µM)
1.32	0.7418	Control	0.6526	Control	19.91033
0.66	0.6737	EGCG 10	0.6971	S 10	19.95483
0.33	0.6309	EGCG 25	0.7184	S 25	19.97613
0.18	0.5727	EGCG 50	0.7505	S 50	20.00823
0.09	0.5835	O-EGCG 10	0.6895	P 10	19.94723
0.045	0.5709	O-EGCG25	0.7003	P 25	19.95803
Blank	0.596	O-EGCG 50	0.782	P 50	20.03973

**Table 3:** Effect of EGCG on lipid peroxidation (TBARS formation).

Standard Concentration	Absorbance	Sample	Average Absorbance	Standard Deviation
0	0	Control	0.249	0.018
0.5	0.017	EGCG 10	0.256	0.023
1	0.032	EGCG 25	0.355	0.016
2	0.071	EGCG 50	0.355	0.015
4	0.141	O-EGCG 10	0.277	0.019
6	0.212	O-EGCG25	0.299	0.024
		O-EGCG 50	0.425	0.040

**Table 4:** Effect of EGCG and O-EGCG on inflammatory cytokines (IL-6, IL-1β, TNF-α).

Sample	IL-1β	IL-6	TNF-α
	Absorbance ± SD		
Control	0.053 ± 0.007	0.135 ± 0.010	0.025 ± 0.001
EGCG 10	0.022 ± 0.005	0.109 ± 0.003	0.009 ± 0.003
EGCG 25	0.017 ± 0.003	0.104 ± 0.004	0.007 ± 0.001
EGCG 50	0.005 ± 0.001	0.091 ± 0.006	0.004 ± 0.002
O-EGCG 10	0.012 ± 0.001	0.095 ± 0.006	0.009 ± 0.004
O-EGCG25	0.007 ± 0.003	0.087 ± 0.002	0.007 ± 0.001
O-EGCG 50	0.002 ± 0.001	0.079 ± 0.009	0.005 ± 0.001

**Table 5:** Molecular docking score, interactions, and bond length of theasinensin A and epigallocatechin gallate compounds for anti-inflammatory markers and M^pro^ of SARS-COV2.

Compound (PubChem ID)	Docking Score (kcal/mol)	H-Bonds	Bond Length (Å)
***Interleukin-1 (IL-1)***			
Theasinensin A	−8.9	H80-A:SER13:O	4.27
		A: SER13:HG-N:O25	2.91
		H41-A:ASN107:OD1	4.88
		H39-A:GLN126:OE1	5.57
		H39-A:ASP145:OD1	4.78
		B:ARG163:HH11-N:O36	4.93
		B:ARG163:CD-N:O34	4.91
EGCG	−8.3	H34-A:GLU105:OE2	2.37
		H34-A:ASN108:O	2.45
		H37-A:MET148:O	2.25
		A:GLN149:HE21-N:O1	2.64
		H-A:PHE150:O41	1.96
		C2-B:ASN204:OD1	2.95
		B:ASN204:HD21-π	2.56
***Tumour necrosis factor-alpha (TNF-α)***			
Theasinensin A	−8.4	A:TYR119-π	3.87
		H77-A:LEU120:O	3.10
		A:LEU120:CO-π	3.95
		H45-A:GLY121:O	2.49
		H46-A:GLY121:O	3.09
		H45-B:TYR151:OH	2.59
EGCG	−7.2	H20-A:ILE58:O	2.18
		A:GLN61:OE1-NO38	2.89
		A:TYR119-π	3.97
		B:SER60:HN-O34	2.85
		H37-B:TYR151:OH	2.12
***Interleukin-6 (IL-6)***			
Theasinensin A	−7.2	A:ARG30:HH11-O69	5.79
		H39-A:ASP34:OD1	3.98
		A:GLN175:HE21-O77	4.64
		A:GLN175:HE22-O14	4.50
		H45-A:GLN175:O	4.35
		A:ARG179:HE-O46	5.42
		A:ARG179:HH21-O44	3.28
		A:ARG182:HH22-O75	5.89
		A:ARG182:HH22-H33	2.51
EGCG	−6.4	A:ARG30:HH11-π	3.92
		H33-A:ARG30:O	4.68
		H35-A:ASP34:OD1	4.96
		H25-A:ASP34:OD1	4.36
		H23-A:ASP34:OD1	3.05
		A:SER37:CB-O22	3.14
***Main protease (M^pro^) of SARS-CoV-2***			
Theasinensin A	−8.70	A:HIS41:HE2-O60	4.64
		A:MET49:SD-π	7.51
		H80-A:PHE140:O	4.90
		H82-A:LEU141:O	6.12
		A:ASN142:HD21-O36	5.25
		A:CYS145:SG-O58	3.65
		A:CYS145:SG-π	5.37
		A:HIS163:HE2-O81	2.15
		H76-A:MET165:SD	2.44
EGCG	−7.77	H41-A:THR190:O	2.64
		H34-A:LEU141:O	2.25
		A:ASN142:HN-O22	2.84
		H37-A:ASN142:OD1	2.50
		A:MET165:SD-π	5.13
		A:GLU166:OE1-π	3.99
		H41-A:ARG188:O	2.18

## Data Availability

Data are contained within the article or supplementary material.

## Supplementary Materials

The following supporting information can be downloaded at: https://www.mdpi.com/article/10.3390/antiox11020294/s1, Table S1: Binding site and grid box of the IL-I, IL-6, TNF-α of human and Main protease of SARS-CoV-2.
