# Supplementary material for: Assessment of Antioxidant, Immunomodulatory Activity of Oxidised Epigallocatechin-3-Gallate (Green Tea Polyphenol) and Its Action on the Main Protease of SARS-CoV-2—An In Vitro and In Silico Approach
Source: Antioxidants (Basel). 2022 Jan 31;11(2):294. doi: 10.3390/antiox11020294 (PMC8868081; doi:10.3390/antiox11020294)
Supplement: Supplementary file 1 [file antioxidants-11-00294-s001.zip › antioxidants-1552694-supplementary.pdf]

Supplementary table

Table S1. Binding site and grid box of the IL-I, IL-6, TNF-α of human and Main protease of SARS-CoV-2.

S. No	Name of the protein	Grid box			Binding site residues
		X	Y	Z	
1	IL-1	37.40	11.60	14.92	Chain A: Arg11, Gln14, Gln15, Lys27, Gln32, Gly33, Gln34, Glu128
					Chain B: Ala1, Agr4, Glu51, Lys93, Lys94, Glu105, and Asn108
2	IL-6	-6.86	-13.37	0.2	Gln175, Arg179, Glu178, Arg182,
3	TNF-α	-18.62	74.43	42.05	Chain A: Leu57, Tyr59, Ser60, Tyr119, Leu120, Gly121, Tyr151
					Chain B: Leu55, Leu57, Tyr59, Ser60, Tyr119, Leu120, Gly121, Tyr151
4	Main protease	-10.28	12.57	68.21	Thr24, His41, Phe140, Leu141, Asn142, Gly143, Cys145, His163, His164, Met165, Glu166, Pro168, Gln189, His172, Gln189, Thr190, Ala191, Gln192
